# Books Review

**Published:** 1870-06

**Authors:** 


### Books Review.

*The Cell Doctrine. Its History and Present State.* By James Tyson, M. D.

This work appears opportunely for the American public, and from a somewhat careful examination of the book we see that it treats of its subject in a worthy manner. A history of the " cell doctrine," occupies the greater portion of the book, and is given by the author with the faithfulness and impartiality of a historian. In the collection of facts, the author has endeavored to attain completeness; he has presented appropriate illustrations as they are needed, and has added a bibliographical list of published works on the topic. The history of the subject is brought down to the very date of the publication of the work, in the conclusion of which, the author presents his own views, as resulting from a careful examination and comparison of the views of other observers, as well as from his own personal observation, which, as lecturer in microscopy in two professional schools in Philadelphia, has been somewhat extended. The book is published in good style, is inexpensive, and will be valuable to those who are interested in the subject of the development and growth of organisms, a matter which so strongly agitates the scientists of the day. Dr. Tyson has stated his views briefly thus:

In conclusion, then, it may be stated, 1st, that the "cell," or "elementary part," originating only in a pre-existing cell, is the ultimate morphological element of the tissue of animals and plants.

2d. That the cell, contrary to the belief of the earlier histologists, and, indeed, many later observers, is *rarely vesicular* in its structure, but generally more or less solid throughout.

3d. That the cell is composed of "germinal" or living matter which is central, and includes "nucleus," "endoplast," "protoplasm" and "sarcode;" and of "non-germinal," or "formed" matter, which is peripheral, and corresponds with "cell wall" and "intercellular substance."

4th. That this germinal matter of the cell in a part or all of its substance, may assume a special morphological state, usually round or oval, commonly known as the "nucleus" of the cell, which, when present, is always a young center of germinal matter; but that in other instances both animal and vegetable cells may be complete without this special form of germinal matter or "nucleus," as in the non-nucleated amœbæ and protogenes primordialis of Hæckel, the non-nucleated monads of Cienkowsky, and in the leaf of Sphagnum, in such Algæ as Hydrodictyon, Vaucheria, and Caulerpa, and in young germinating ferns.

5th. That in consequence of these facts, it cannot be said that in the nucleus alone resides the power to reproduce the cell, since we find the nucleus not essential, but that in the germinal matter, of which after all, the nucleus, when present, is but a part, resides this function.

6th. That when the smaller body within the nucleus, usually known as the "nucleolus," is present, and as it often is in complete cells, it is simply a younger center of germinal matter than is the nucleus itself, and is the last formed portion of germinal matter, instead of being the oldest part of the cell, as originally taught by Schleiden and Schwann. And thus, according to the latest views, the whole process is reversed. The old order of succession being 1st. The "nucleolus;" 2d. About this the "nucleus;" and finally about this the "cell wall," which embraces the cell contents. Now, however, what constitutes the "cell wall" when present, is the oldest part of the cell; next in age are the socalled "contents," whether germinal matter or not; next the "nucleus," and last and youngest the "nucleolus."

7th. That the formed material constituting cell wall and intercellular substance may be somewhat chemically different from the germinal matter, or protoplasm whence it was converted, as the secretions of gland-cells, or may be a simple condensation of the exterior of the cell, as in the red blood disc.

8th. That the so-called "free nuclei," so often referred to by pathologists in their descriptions of minute structures, are simply masses of germinal matter, smaller than those to which the name cell is usually given, which, if time be permitted, will pass into perfect cells by the usual production of formed matter on their periphery; that they do not originate spontaneously, but from previously existing germinal matter. So, too, "granules," if they be composed of germinal matter, present the same attributes and endowments, arising from previously existing germinal matter, capable of growing, multiplying, and assuming all the characters of fully formed cells, but never originating spontaneously. Granules otherwise composed are *histolytic* and *not histogenetic*,—that is, they result from the breaking down of tissue rather than go to building it up.

---

*Modern Therapeutics: A Compendium of Recent Formula and Specific Therapeutical Directions.* By GEO. H. NAPHEYS, A. M., M. D. Philadelphia: S. W. BUTLER, M. D.

The author has collated from a variety of sources, such formulæ and methods of treatment as are now in use by the best medical authorities, and after having presented them in a series of articles in the *Philadelphia Medical and Surgical Reporter*, now offers them in book form to the profession. He remarks that while books of similar character have previously been arranged with reference to the articles of the Materia Medica, in the present work the nosological order has been adopted and effort has been made to assign proper rank to Therapeusis. Another volume is hereafter to be published, which is to add completeness to work already accomplished.

---

*Williams on Disease of the Eye.* Boston: FIELDS, OSGOOD & Co.

We are happy to acknowledge the receipt of the third edition of this most excellent work upon the diseases of the eye. While it does not claim to contain all that is known of the eye, or aspire to take the place of the larger and more complete works, it still comprises a plain and very complete description of the common diseases and accidents which are liable to befall the organ of vision, and is sufficiently comprehensive in its scope to afford the general practitioner a satisfactory guide. It is, indeed, better adapted to the wants of the physician in general practice, than any of the elaborate complete works which require thorough education and much time to fully appreciate their minute teachings. It is important that every physician be educated to some extent in Ophthalmology; at the present time, no student should graduate without having paid adequate attention to this subject, and no College should be recognized which does not make provision for teaching it.

It is not, perhaps, to be expected, neither is it desirable that all general practitioners become experts in Ophthalmology, but it is of infinite importance that they become somewhat intelligent on the general subject, and understand at least enough to know what cases they may safely advise, and what they are wholly unqalified to treat; from lack of this, many a poor victim is to-day suffering from hopeless blindness. This work by Dr. Williams, now very extensively known in the profession, is eminently adapted to the wants of students and general practitioners. Its plain terms, faithful and clear description of disease, and condensed form, commend it to the favorable consideration of the profession. It explains and illustrates the theories of refraction and accommodation, the development of which constitutes so much of the recent advances in Ophthalmology. The methods of making Ophthalmoscopic examinations are described and illustrated, and the common diseases and accidents of the eye are described plainly and fully, making it in all respects complete so far as consistent with its general design of brevity.

---

*Twenty-seventh Annual Report of the Managers of the State Lunatic Asylum at Utica, for the year* 1869.

In order to be convinced that a Lunatic Asylum is not merely an institution for the physical coercion of such human beings as are without reason, but is rather an hospital for the medical and surgical treatment of diseases of the nervous system, one may well read the recent reports from such institutions; physicians can hardly fail to learn something worth the knowing from the yearly hospital reports of the medical officers who have this class of diseases in trust. The whole number of patients treated at the Utica Asylum since its opening is 9,225, and of these, 3,572 recovered, and 1,407 were discharged, so far improved as to return to ther usual duties. The Managers state, that from long experience and observation, they cannot too strongly urge that when insanity is clearly recognized, the patients should be promptly sent to an asylum. The reluctance of friends, and sometimes unreasonable and unfounded prejudice against asylums, too often prevent admission in the early stages of insanity. It ought to be considered that delay often results in confirmed insanity, and not unfrequently in death. Dr. Thurman, a distinguished writer on the subject, states that if cases were treated within three months of the first attack, four-fifths would recover, but if twelve months elapsed, four-fifths were incurable, and so in proportion as the time was longer or shorter. Attention is called to the condition of the insane confined in county poor houses, and the necessity of enlarged asylum accommodations, and it trusted that the liberality of the State will soon provide for the proper security and treatment of the insane. The able and excellent Superintendent, Dr. George P. Gray, in his report, which is a valuable contribution to the scientific study of disease, states that if the large number of patients admitted this year, the table of causation will show that the insanity is directly due to physical causes; no moral cause can be potentially operative, except as developing bodily disorders somewhere in the organism. Insanity is a pathological state of the physical man; the psychological, mental, or psychopathic element is the result of this pathological condition. Very many elements enter into a tabulation of the causation of insanity, and it is necessary that the etiology of each individual case be accurately diagnosed, and treatment appropriate to that case adopted. It is to medicine proper that we are to look for relief from insanity; and moral appliances secondarily receive attention. Griesinger says: There are in affections of the brain, only three distinct categories of morbid elementary symptoms, viz, those of lesions of sensibility, lesions of motion, and lesions of intelligence. The mental or psychic symptoms may also be brought within an equally narrow compass, viz, excitement, depression, and enfeeblement. Last year (Dr. Gray) recommended the appointment of a special pathologist to make such investigations as are now demanded by medical science, but which could not be made as they should be without increasing the medical staff. He feels abundantly satisfied that this was not only a step in the right direction, but that in the appointment by the managers, of Dr. E. R. Hun, of Albany, as special pathologist, the proper person has been secured to carry out the work in this field of scientific inquiry; a task in which not only patient labor is required, but also the sympathy of the medical profession and of the public, and the co-operation and encouragement of State officials, from which sources they have thus far received cordial approval. Their labors, so far, have been confined mostly to experiments with the sphygmograph on the pulse, and to microscopic examinations of morbid tissues. He trusts they may be able to include as they advance: 1. Examinations of secretions in all stages of the disease. 2. The pulse under the sphygmograph to determine its force and character, and whether any, and if so, what coincident relations its various phases may bear to physical states and psychological manifestations. 3. The pulse under the sphygmograph to show the influence of medicine on the circulation. 4. Examination with the ophthalmoscope to ascertain the relations of morbid changes in the optic nerve, vessels, etc., of the eye, to pathologic conditions of the brain and its membranes. 5. The skin, its temperature, color, elasticity, sensibility, etc., in the several forms and stages of the disease. 6. *Post mortem* appearances generally and microscopically. 7. Photographic representations of morbid conditions and specimens. In July last Dr. Henry D. Noyes, of New York, spent some days at the Asylum in the ophthalmoscopic examination of the eyes of patients, giving instruction in the use of the instrument, and pointing out physiological and pathological appearances. This brief clinic was most interesting, highly instructive and useful. In cases of paresis, of which he examined a number, embracing all stages of the disease, he found in each case a lesion of the optic nerve and vessels, characteristic of each stage of the disorder. If paresis, as Calmeil and others maintain, has its origin in chronic cerebral-meningitis, the study of its early symptoms is of the highest importance, and from what he has already seen he feels warranted in asking the attention of medical men to this point ot inquiry. Dr. Gray alluded to another most interesting and important question of research,—that of the relation of phthisis to brain disease; he remarks also upon the sphygmograph and his experience in its use. The work concludes with a full clinical report of a number of different cases of interest which there came under observation. The State is to be congratulated on the prosperous condition of the Utica Asylum, and the ability of the officers who have it in charge.

---

*Archives of Ophthalmology and Otology.* Volume 1. No. 1. By Prof. H. Knapp, M. D., of New York, and Prof. S. Moos, M. D., of Heidelberg. New York: Wm. Wood & Co.

This publication, which is to be issued simultaneously in English and German is of an eminently able and scientific character. The labor expended in its production is of great magnitude, both in the work of preparing its articles, which are altogether original, as well as in the manner of its publication, which is unexceptionable, and its lithographic illustrations, which are numerous and excellent. Three hundred and sixty-four pages are presented in this first issue, which it is intended shall be regularly succeeded every six months by other similar numbers. The articles in the first number are: Entoptic Phenomena connected with the circulation of the blood, by B. A. Pope, M. D., of New Orleans; extirpation of the Fibro-Cartilage of the upper eyelids for the cure of certain cases of Entropion and Trichiasis, by B. A. Pope, M. D., of New Orleans; Test-pipe for Astigmatisn, by Dr. Orestes M. Pray, of Brooklyn; on a modification of Iridectomy Forceps, by Dr. Liebreich, of Paris; modification of the advancement of the muscle, by Dr. R. Liebreich, of Paris; two cases of extraction of foreign body from the Corpus Vitreum, by R. Berlin, of Stuttgart; dislocation of the Crystalline into the Corpus Vitreum and afterwards into the Anterior Chamber, by Henry D. Noyes, of New York; stricture of the Nasal Duct, by E. Williams of Cincinnati; two cases of inflammation of the Tympanum, with development of Polypus, by James Hinton, of London; Embolism of a Branch of the Retinal Artery with Hemorrhagic Infaretus in the Retina, by H. Knapp, of New York; on the formation of Cysts in the Iris, by L. Wecker of Paris; Cataract extraction operations, by Henry W. Williams, of Boston; reports and remarks upon a third series of one hundred cases of Cataract Extraction by the Peripheric-Linear method, by H. Knapp, of New York; alterations of Taste and Sensibility by the application of an Artificial Tympanum in a case of Large Perforations in both Membranæ Tympani, by S. Moos, of Heidelberg; Isolated Rupture of the Choroid, resulting from concussion of the eyeball, with chromo-lithographs, by H. Knapp of New York; on the theory of Binocular Vision, with chromo-lithographs, by H. Kaiser, of Dieburg; contributions to the Pathology of Burns of the Cornea from Lime, with chromo-lithographs, by H. DeGouvea, of Rio de Janeiro; the galvanic reaction of the Nervous Apparatus of Hearing, in conditions of Health and Disease, by W. Erb, of Heidelberg; investigation on the relation between the Handle of the Malleus and the Membrana Tympani, with chromo-lithograph, by S. Moos of Heidelberg; two fatal cases of Ear Disease in Court, by S. Moos of Heidelberg; on the Medico-Legal significance of Atrophy of the Tympanum, produced by hardened cerumen, by S. Moos of Heidelberg; peculiar disturbances of hearing after Cerebro-Spinal Meningitis, and considerable improvement by the galvanic current, by S. Moos of Heidelberg; on Retinitis Leucœmica, by O. Becker, of Heidelberg; a case of Pyœmia from suppurative inflammation of the cavity of the Tympanum, induced by the use of Weber's Nasal Douche, by D. B. St. John Roosa, of New York. We are happy to see so many valuable contributions thus brought together, of which a good number are by American authors. We trust that German industry, American enterprise, and the professional ability of both countries may unite in securing the continued success of this publication.

---

*Relaxation of the Pelvic Symphyses during Pregnancy and Parturition.* By FREDERICK G. SNELLING, M. D.

In a paper read before the Medical Journal Association of New York on "Relaxation of the Pelvic Symphyses," by Frederick G. Snelling, M. D., the author cites two cases which came under his own care and observation, presents accounts of cases by various physicians and authorities, and also describes the anatomical features of the condition of relaxation. After giving the views which have been entertained by other writers he remarks: "I think it is not forcing a conclusion to regard it as proven from what has been advanced that an uncertain, varying degree of relaxation or *ramollisment* does obtain in a very large number of cases in the pregnant and puerperal condition, of a physiological and benign character, and entirely consistent with health, and that it is to the excess alone of this condition that pathological results are due."

"Relaxation is not the only diseased condition of the pelvic articulations incident to pregnancy, nor by any means the gravest; *suppurative* inflammation, with its attendant dangers, frequently sets in, and carries off the patient in spite of all that care and skill can do; and furthermore, *rupture* of the symphyses may take place as a crowning result. The latter usually occurs after a severe labor, and is caused by some disproportion between the fœtal head and the pelvis of the mother, or in some cases by one of the forms of mal-presentation. It generally takes place in osteo-malaceous or rachitic pelves, where the conjugate diameter is greatly shortened. It may take place in either the pubic or sacro-iliac articulation, but its favorite seat is the right sacro-iliac. It has been known to involve two symphyses at once."

Remarks are subjoined which were made at the Association Meeting by Professors Fordyce Barker and Isaac E. Taylor, of whom the former thinks that "the œdema and consequent laxity of the ligamentous tissues may be due to the mechanical obstruction of the venous trunks by the pressure of the presenting part, or fœtal head." Dr. Taylor has the impression that the relaxation of the pelvic, and especially the pubic, is only a part of a physiological process, and that this relaxation sometimes occurs two or three times during pregnancy. The profession will find the whole subject quite fully presented and discussed in this pamphlet, which is a reprint from the *American Journal of Obstetrics* of February last.

---

*Gray's Anatomy, Descriptive and Surgical.* Fifth Revised Edition. Philadelphia: H. C. LEA.

This work is too well known and appreciated to need any extended remarks or criticism. The present American edition has passed through the press under the supervision of Dr. Robert J. Dunglison, who has carefully corrected any errors that may have escaped the hands of its English editor, the well known Dr. T. Holmes, M. A., who remarks that "In this edition the plan of the work has been so far altered that the portion on General Anatomy, which was previously scattered through the book, has been collected into an introductory chapter, and re-written so as to furnish the student with a very succinct, but it is hoped, sufficient introduction to the study of Microscopic Anatomy; and to this has been added a short description of the chief processes of the development of the ovum, and of the structures characteristic of the fœtal state; a subject which, though undeniably an integral portion of Human Descriptive Anatomy, was passed over in previous editions." The illustrations in this chapter on General Anatomy have been borrowed principally from Koelliker, Todd & Bowman, and Harley & Brown. There are, in short, fifty additional pages, and sixty-seven new illustrations, introduced to the present work. In the descriptions of the minute structure of the different tissues of the body, such statements are given as may be plainly understood, are for the most part generally admitted, and are such that the student, with the microscope, may demonstrate them for himself. This description of the structure of the various tissues which have been distinguished in name in the work, but have been hitherto undefined by it, cannot but supply a real need of such students as are not possessed of special works on Histology.

---

*Reports of the Trustees and Superintendent of the Butler Hospital for the Insane, at Providence, R. I.*

This institution, founded by private munificence, is under the charge of John W. Sawyer, M. D. The buildings are situate at a convenient distance from the noise and dust of the city, and we have reason to believe that its officers are able and efficient in their ministration to its necessities and comforts.

*A Manual of Clinical Medicine and Physical Diagnosis.* By THOMAS HAWKES TANNER, M. D. Philadelphia: H. C. LEA.

As the demand for this work, which was first issued fifteen years since, still continued; some time after it was out of print, the author entrusted to an editor, Dr. Tilbury Fox, the task of preparing this second edition, by whom many additions have been made. The Laryngoscope, Ophthalmoscope, Sphygmograph, and Thermometer, have been made the subject of special sections; Pericardial, Endocardial, Abdominal, Cerebro-Spinal, and Feigned Diseases, are also given particular attention. Other marks of progress might be noted, and on the whole, the book ranks high among works of this class.

---

*Report of the Resident Physician of Brigham Hall, an Hospital for the Insane, for* 1869.

This private institution is situate in Canandaigua, and is under the judicious management of George Cook, M. D. His report says that the total number of recent cases treated in the hospital in the course of the year, is forty-four; of this number thirty have been discharged recovered, two unimproved, two have died, and eight remained under treatment at the close of the year. Of these eight cases two are, at the time of writing the report, quite well, while in four others, convalesence is so far established as to indicate with certainty, their complete restoration. This would make thirty-six recoveries in forty-four cases, with four of them remaining under treatment too short a time to determine the result. No array of facts or opinions would be more conclusive on the importance of early hospital treatment in insanity.

---

*Transactions of the Medical Association of the State of Missouri.*

This society having been thoroughly reorganized, has now published a report of its proceedings from the date of its reorganization, December 10th, 1867, to the annual meeting, April 27, 1869, inclusive. We notice in the volume interesting addresses by Drs. M. A. Pallen, Montgomery, and J. S. Moore; also reports respecting the memorial presented the legislature on the qualification which practitioners of medicine in the State should possess; also the memorial to the American Medical Association respecting a higher standard of scholarship necessary to a degree. In the Appendix to the Report of Transactions, may be found valuable essays, entitled: Fœticide or Criminal Abortion, by M. A. Pallen, M. D.; statistics of fifty-one successive capital amputations performed on fifty individuals, by A. Hammer, M. D.; causes and relationship of some hereditary diseases, by W. B. Outter, M. D.; advancement of knowledge of diseases of females during the last quarter of a century, by G. M. B. Maughs, M. D.; improved Hodgen Splint for treating fractures of the Femur, by E. A. Clark, M. D.; fractures of the Olecranon, by E. A. Clark, M. D.; Suspension Splint of treating fractures of the leg, by E. M. Clark, M. D.; on the treatment of Lachrymal Obstructions, by John Green, M. D.; use and abuse of the Obstetric Forceps, by G. M. Maughs, M. D.; Artificial Pupil, by William Dickinson, M. D., illustrated by two lithographic plates, giving test diagrams for the detection and measurement of Astigmatism.

---

*Thirteenth Annual Report of the Trustees of the State Lunatic Hospital at Northampton, Mass.*

This institution, which has as fine a bu'lding and location as could well be found, is under the immediate care of Pliny Earle, M.D., a gentleman of culture and well-known efficiency.

---

*Sixteenth Aanual Report of the Trustees of the State Luna'ic Hospital at Taunton, Mass.*

The Superintendent, Geo. C. S. Choate, M. D., here presents carefully prepared statistical tables respecting the management of the institution, which is one of three hospitals for the Insane of the State of Massachusetts, which are situate in Worcester, Taunton, and Northampton respectively.

---

*Second Annual Meeting of the Nebraska State Medical Society.*

We have received several copies of the *Omaha Daily Republican* giving an account of the proceedings of the State Medical Society of Nebraska at its second annual meeting, which was held June 8th and 9th, at which Dr. N. B. Larsh was elected President, and Drs. J. C. Denise and S. D. Mercer, Secretaries. A spirit of good-will and zealous devotion to the science of medicine seems to pervade the members of the Society.

---

*Nocturnal Enuresis and Incontinence of Urine.* By FREDERICK G. SNELLING, M. D. New York: TURNER & MIGNARD.

After naming an extended list of primary causes of the disorder treated of, the author states: "In glancing over the list of causes, it is apparent that diverse as these may be, they can only act by giving rise to one of three prime conditions, viz, Atony, or paralysis of the bladder, itself, permitting over-distension, and resulting in stillicidium; abnormal irritability and contractility of the bladder, sufficient to overcome the resistance of the sphincter; or atony, or paralysis of the sphincter vesicae itself." We may have insufficiency of power, or actual paralysis, either of the cerebro-spinal fibres, of the sympathetic system, or of the muscular coats of the bladder itself. Manifestly, if the deficiency be in the voluntary fibres, it would be worse than folly to expect a child to control his sphincter muscle, but I am convinced that this is rare in children to the extent of actual paralysis. There is generally a combination of feebleness of innervation, and a habit of inattention preserved from babyhood. The *nervous system may become habituated to inattention*, as also on the other hand to the unconscious exercise of voluntary power. In the incontinence of the old, or middle-aged, the will is not often at fault; there we have to deal with a condition of actual paralysis, or irritation, entirely distinct from the exercise of voluntary power, dependent upon different causes, and requiring different treatment. The mutual relations and possible interactions of these different lesions, should be steadily kept in view. All treatment will prove unavailing, or at best only palliative, until the exciting cause, or *point du depart* of the disease is discovered."

The treatment deemed appropriate by the writer for these different classes of lesion, is given at length in the pamphlet, which may be obtained of the publishers.

